# The Outcomes of Flexor Tendon Injury Repair of the Hand: A Clinico-Epidemiological Study

**DOI:** 10.7759/cureus.33912

**Published:** 2023-01-18

**Authors:** Vikrant Ranjan, Milind Mehta, Mugdha Mehta, Parijat Mishra, Tulika Joshi, Tushar Kumar

**Affiliations:** 1 Plastic and Reconstructive Surgery, Rajendra Institute of Medical Sciences, Ranchi, IND; 2 Plastic Surgery, Smt. NHL Municipal Medical College, Seth KM School of Postgraduate Medicine and Research, Ahmedabad, IND; 3 Medicine, B.J. Medical College, Ahmedabad, IND; 4 General Surgery, Rajendra Institute of Medical Sciences, Ranchi, IND; 5 Fetal Medicine, Berlin Diagnostics and Day Care, Ranchi, IND; 6 Anaesthesiology, Rajendra Institute of Medical Sciences, Ranchi, IND

**Keywords:** joint contracture, post-operative joint infection, neuro vascular repair, epitendinous repair, physiotherapy rehabilitation, flexor tendon rupture

## Abstract

Background and objective

The incidence of flexor tendon injury is estimated to be 7-14 per 100,000 population. In India, such injuries are common and about 5% of these injuries require repair of the flexor tendon. In the present study, we share our experience of hand flexor tendon repair at a tertiary care center in western India.

Material and methods

Over a period of three years, 45 patients were admitted for tendon repair. After performing a proper evaluation, patients were taken for tendon repair. Primary outcome and secondary outcome parameters were assessed at the end of three months. Physiotherapy was continued for a longer duration in patients with movement restrictions. Data were compiled at each stage.

Results

The mean age of the patients was 28.84 years (range: 13-68 years) with a majority of the cases belonging to the age group 15-60 years. The majority of hand injuries were accidental (caused by work-related accidents, machine injuries, or animal bites) amounting to 80% (n=36), followed by assault cases (11%, n=5) and self-inflicted injuries, i.e., attempted suicides (around 9%, n=4). Among all injuries, the majority were in zone V (60%, n=27) followed by 24.4% (n=11) of cases in zone II. A few cases were in zone I, III, and IV (2.2%, 11.2%, and 2.2% respectively). The Buck-Gramcko scoring for primary injury was excellent with a recovery rate of 57.78%.

Conclusion

Flexor tendon injuries should be repaired with the aim of recovering strength as well as mobility. For optimal outcomes, total active motion protocol should be commenced immediately after the surgical repair. However, long-term physiotherapy may be required for attaining desired benefits.

## Introduction

It is generally proposed that the hominid lineage began when the quadrupedal stance changed to bipedal, letting the hands become the executioner of all the daily activities. Consequently, as the human executing organ, the hands also became more prone to injury and overuse. The incidence of flexor tendon injury is estimated to be 7-14 per 100,000 population [1,2]. The data from the Indian subcontinent is limited, but as per one hospital-based study, repair of flexor tendon was needed in 5% of new hand surgery cases [3]. While repairing a flexor tendon, it should be kept in mind that it is a complex unit consisting of the tendon, tendon sheath, and pulley. The aim of the repair should be the restoration of tendon strength with the achievement of smooth tendon gliding in the narrow pulley system. In the present study, we share our experience of hand flexor tendon repair at a tertiary care center in western India. Our primary objective was to study the outcomes following flexor tendon injury repairs of the hand. The secondary objective was to evaluate the demographic pattern of flexor tendon injuries and to assess the incidence of flexor tendon injuries of the hand.

## Materials and methods

This was a prospective observational study that was approved by the Institutional Ethics Committee (IEC No: 19/112 dated 19.09.2016). The inclusion and exclusion criteria are presented in Table 1.

**Table 1:** Inclusion and exclusion criteria

Inclusion criteria	Exclusion criteria
All patients with flexor tendon injuries of the hand (in any zone and finger, with any associated neurovascular and/or bony injury) admitted during the period from October 2016 to February 2019	Patients with flexor tendon injuries but who had not completed a full course of treatment
All patients with open hand flexor tendon injuries	Patients with closed-hand tendon injuries
Patients with flexor tendon injuries who were initially treated elsewhere and referred to our hospital for further management were included. Consent from the patient was required, or from a guardian, if the patient was minor	Patients with finger amputations
	Patients with crush injuries of the hand
	Patients requiring tendon graft

Data were collected prospectively from October 2016 to February 2019 for consecutive nonrandomized patients presenting to our tertiary care center in western India. A total of 45 patients were included in the study. All the patients presenting to the emergency department with tendon injuries who were advised admission and surgery in the aforementioned time frame were included. For the purposes of analysis, a limitation in the range of motion in any finger on examination or cut and visible ends of the tendon were considered to be a case of tendon injury. Patients refusing intervention at any time during the course of treatment and patients with closed tendon injuries were excluded from the study.

After obtaining consent, all the included patients were subjected to detailed history-taking including demographic data (age, sex, and occupation) with special attention to the length of hospital stay, presence of concomitant medical problems (defined as medical problems that might alter the course of recovery from the tendon repair, e.g., diabetes, chronic obstructive pulmonary disease, heart disease, asthma), injury etiology, mode, motive, etc. A detailed local examination of tendon injury was done on all the fingers including the hand and the data were recorded. The degree of flexion was investigated at each joint and the extent of functional impairment was recorded. Investigations with respect to preoperative fitness such as complete blood count, renal function tests, serum proteins, liver function tests, chest X-ray, and electrocardiogram wherever necessary were done in addition to X-rays of the local parts. Orthopedic consultation was done as part of the protocol to rule out any bony injuries and to determine if any interventions were needed from their side.

Patients were taken to the operating room for tendon repair depending on their fitness. Preoperative Doppler assessment and CT angiogram were advised for patients with suspected vascular injury. Peripheral vessels (radial and ulnar artery) repair was done by us along with the nerve repair, under loupe magnification with monofilament polypropylene 8-0/7-0. Tendon repair was done with monofilamentous polyamide reverse cut needle 4-0 as core and 5-0 for epitendinous repair (interrupted). The modified Kessler technique [4] was used in most of the cases as a protocol. In some of the cases of zone 2 flexor tendon injury, only FDP repair was done for the smooth gliding of tendons. In flat tendons, the figure-of-8 or mattress sutures were placed. Kleinert was applied in the distal tip of fingers in flexor tendon injuries with silk sutures. A postoperative splint was applied with plaster of Paris. The thumb splint was applied separately whenever required. The modified Kleinert [5] protocol was taught to the patient and encouraged to follow the same. The dressings were checked on alternate days and patients were discharged after two dressings if the condition was found satisfactory. Patients with the associated neurovascular repair were discharged at a later date.

Suture removal was done after 14 days. Kleinert and splint removal was done after 24 days with follow-up physiotherapy under guidance. Follow-up was done after three weeks and six weeks, and the final follow-up was done at three months. Primary outcome and secondary outcome parameters were assessed at the end of three months [6]. The range of motion was calculated using a goniometer and grasp strength was calculated using a hand-held dynamometer. Physiotherapy was continued for a longer duration in patients with movement restrictions. Data were compiled at each stage. Patients were followed up for three months following discharge and details pertaining to long-term complications, contractures, and compliance with physiotherapy were noted.

Statistical analysis was done using IBM SPSS Statistics (IBM Corp., Armonk, NY). All the data collected were presented as mean and standard deviation (SD) or percentages wherever applicable.

## Results

Over the three-year study period, there were 45 cases of flexor tendon injury of the hand. The mean age of the patients was 28.84 years (range: 13-68 years) with a majority of the cases belonging to the age group 15-60 years. There was a preponderance of male cases (n=39) over female cases (n=6; male-to-female ratio: 6.5:1). The majority of patients (55.5%) were manual laborers, followed by students (26.6%), field workers or farmers (15.5%), with housewives comprising a very low proportion (4.4%). Comorbidities such as hypertension and diabetes were observed in a few cases but a large number of cases reported having tobacco addiction in inhalational or oral form. All the cases with self-inflicted wounds were sent for psychiatric evaluation. Table 2 presents the demographic profile of the patients.

**Table 2:** Demographic profile of the patients

Variable	Number (n)	Percentage (%)
Age (in years)		
<15	2	4.4%
15–30	24	53.4%
30–60	17	37.8%
>60	2	4.4%
Sex		
Male	39	86.7%
Female	6	13.3%
Occupation		
Worker	25	55.5%
Farmer	7	15.5%
Student	12	26.6%
Homemaker	2	4.4%
Comorbidity		
Hypertension	5	11.1%
Diabetes	2	4.4%
Smoking	27	60%
Oral tobacco	22	48.8%
Psychiatric condition	3	6.6%

Injuries

The majority of hand injuries were accidental (caused by work-related accidents, machine injuries, or animal bites), accounting for 80% (n=36) of cases, followed by assault cases (11%, n=5) and self-inflicted injuries, i.e., attempted suicides (around 9%, n=4). Out of 36 accidental tendon injury cases, 25.4% were machine-cut, 70.1% were domestic-related, and the rest (4.5%) were caused by animal bites. Most of the cases presented early, i.e., within six hours of injury (62.2%). A small portion of cases had a delayed presentation, i.e., after two days of injury (8.8%). The associated injury to the radial or ulnar nerve was seen in eight cases (17.77%), while damage to major vessels was seen in nine cases (20%). Phalangeal fracture and metacarpal fracture were seen in two cases (4.44%). Details of the injuries are summarized in Table 3.

**Table 3:** Injury details

Variable	Number (n)	Percentage (%)
Mode of injury		
A. Accidental	36	80%
Workplace	26	
Domestic	8	
Animal bites	2	
B. Assault	5	11%
C. Self-inflicted	4	9%
Time to presentation since the injury (hours)		
<6	28	62.2%
6–48	14	31.1%
>48	3	6.7%
Associated injuries		
Nerve	8	18.7%
Vessels	9	20%
Bone	2	4.4%

Complications

We noted that the mean hospital stay was 7.8 ±4.6 days from admission to discharge, whereas surgical site infection (SSI) was noted in 13.33% of cases. A few of the patients had finger contractures (4.44%). One patient had to undergo revision surgery and 11.11% of patients had rehabilitation problems. The incidence of complications is shown in Table 4.

**Table 4:** Hospital stay and complications SD: standard deviation; SSI: surgical site infection

S. no.	Variable	Value	Percentage
1	Hospital stay, days, mean ±SD	7.831 ±4.61	-
2	SSI	6	13.33%
3	Revision surgery	1	2.20%
4	Contracture	2	4.44%
5	Rehabilitation	5	11.11%

Outcomes

Primary Outcome

**Figure 1:**
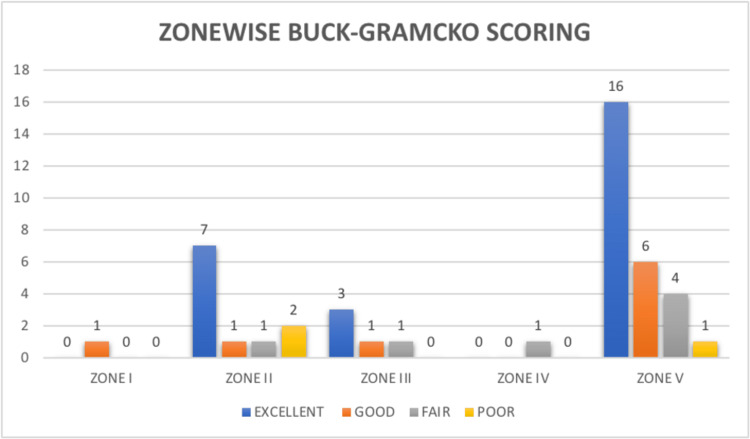
Buck-Gramcko scoring

Among all injuries, the majority were in zone V (60%, n=27) followed by 24.4% (n=11) of cases in zone II. A few cases were in zone I, III, and IV (2.2%, 11.2%, and 2.2% respectively). The flexor tendon repair primary outcome was measured using the Buck-Gramcko scoring system, in which 57.78% of patients showed excellent scores (n=26); 20% of the patients were found to have good scores (n=9), 15.55% of patients (n=7) had fair scores, while 6.67% patients had a poor outcome. The Buck-Gramcko scoring is shown in Figure 1.

Secondary Outcome

The secondary outcome parameter was calculated by measuring the passive range of movement, which was compared with the contralateral normal finger and categorized as normal or decreased against the normal finger. It was found to be normal in 95.6% of injuries. Grasp strength assessed using a hand-held dynamometer was found to be decreased in 24.4% of patients.

Patient Satisfaction

A total of 40 patients (88.9%) were satisfied after flexor tendon repair with the majority having good satisfaction (73.3%) at the end of three months. The poorly satisfied patients were the ones with complications and they were advised to undergo prolonged physiotherapy and regular follow-up in OPD. The outcomes are summarized in Table 5.

**Table 5:** Outcomes

Secondary outcome parameter		Number of cases	Percentage
Passive range of movement	Normal	43	95.5%
	Decreased	2	4.5%
Grasp strength	Normal	34	75.5%
	Decreased	11	24.5%
Return to previous activity	Satisfactory	40	88.9%
	Unsatisfactory	5	11.1%

## Discussion

For the successful repair of the flexor tendon, early mobilization in the healing phase is advocated. The tendon repair not only needs to be fine (not bulky) so that it could slide through the narrow pully system, but it should also be strong enough to enable mobilization. For achieving these outcomes, two types of suture techniques (core sutures and circumferential sutures) are used while repairing the flexor tendons. The main function of core sutures is to provide strength to the repair [7] and circumferential sutures make the repaired junction smooth and also add strength to the repair to some extent [8].

In the current study, there was a male predominance with a male-to-female ratio of 6.5:1. Kumar's prospective study of 43 cases with flexor tendon injury over two years [9] had a male-to-female ratio of 6.17:1, which is very similar to the current study. The phenomenon of males having more injuries may be due to the socioeconomic structure of the society in which males are more exposed to occupational hazards. Wentzel's research study in 2017 on the rehabilitation of 35 cases with flexor tendon injuries documented 42% of cases as unemployed and 42% as unspecified with respect to their occupation. Unlike the current study, where 48% of the patients were manual laborers and 19% were farmers, only 8.5% of cases were in the above-mentioned categories in the study by Wentzel. The difference can be attributed to the different geopolitical backgrounds of the two studies as well as the unlawful working conditions prevailing in some countries. The same study [10] documented adhesion (31.43%) and infection (20%) as major postoperative complications, along with 8.51% cases of tendon rupture and 5.7% contractures. The same complications were noted in the current study, but the incidence was very low in comparison. The incidence of tendon rupture varies from study to study but it is usually below 10% [11]. The development of contractures can be minimized by active pull-through exercise with the wrist in flexion. If the physiotherapy fails, tenolysis surgery is indicated after three to six months of repair [12]. 

In a prospective study by Kumar on the outcome of flexor tendon injuries, 58% of the patients were in zone II, 25% in zone III, 15% in zone V, and 2% in zone I. Similarly, in a randomized controlled trial by Rigó et al. on the outcomes of flexor tendon injury in zones I-III [13], the majority of patients belonged to zone II (68.11%), followed by 26.08% in zone I, and only 5.8% in zone III. When comparing the outcomes of flexor tendon injuries, our results were comparable with those of Saini et al. [14]. In their study, which assessed the effect of early active motion on flexor tendon injury repairs, 62.29% of cases had excellent outcomes and 22.95% had good outcomes. Fair and poor outcomes were seen in 9.83% and 4.92% of cases respectively. This outcome was markedly different from the research conducted by Wentzel [10]. The difference can be attributed to the differences in the ethnic backgrounds of patients, different surgical techniques and rehabilitation protocols, and different criteria of measurement. Newport et al. [15] reported that 95% (57/60) of patients were satisfied with their functional results. Six patients were forced to change their jobs and seven patients had to make changes to their sports activities or hobbies because of the injury.

Limitations of the study

This study has a few limitations. Primarily, this was an observational study. Moreover, the study sample size was relatively small. The treatment of these injuries takes a long time to follow up. Also, there was potential variability in treatment protocols. Flexor injuries of the hand are not common and hence a multi-center study might have yielded stronger conclusions.

## Conclusions

Flexor tendon injuries should be repaired with the aim of regaining strength as well as mobility. Strength can be achieved by increasing the number of strands in the core sutures. For achieving optimal outcomes, total active motion protocol should be commenced immediately after the surgical repair. As discussed earlier, epitendinous sutures and early mobilization minimize the development of contractures in the repair site. However, full recovery may require long-term physiotherapy.
